# Jejunal endoscopic full-thickness biopsy for chronic idiopathic intestinal pseudo-obstruction

**DOI:** 10.1055/a-2689-5678

**Published:** 2025-09-05

**Authors:** Yo Ishihara, Takaomi Kessoku, Hibiki Kanagawa, Hideyuki Chiba, Yuichiro Hayashi, Hirotoshi Ebinuma, Atsushi Nakajima

**Affiliations:** 1 548893 Department of Gastroenterology, International University of Health and Welfare Faculty of Medicine Graduate School of Medicine, Narita, Japan; 2 625200 Department of Palliative Medicine, International University of Health and Welfare Narita Hospital, Narita, Japan; 3 26438 Department of Gastroenterology and Hepatology, Yokohama City University School of Medicine Graduate School of Medicine, Yokohama, Japan; 4 74155 Department of Gastroenterology, Omori Red Cross Hospital, Ota-Ku, Japan; 5 625200 Department of Pathology, International University of Health and Welfare Narita Hospital, Narita, Japan; 6 36755 Department of Gastroenterology, International University of Health and Welfare Atami Hospital, Atami, Japan

A 40-year-old man presented with a 2-year history of recurrent bowel obstruction without mechanical causes. Abdominal radiography and computed tomography demonstrated marked small bowel dilatation, suggesting chronic idiopathic intestinal pseudo-obstruction ( Fig. 1 ). Although surgical full-thickness biopsy is regarded as the gold standard for definitive diagnosis, concerns remain regarding its invasiveness and risk of postoperative adhesions 1 . Endoscopic mucosal resection with an over-the-scope clip (EMRO), initially developed for submucosal lesions 2 , was adapted for jejunal endoscopic full-thickness biopsy (EFTB) ( Video 1 ).

**Fig. 1:**
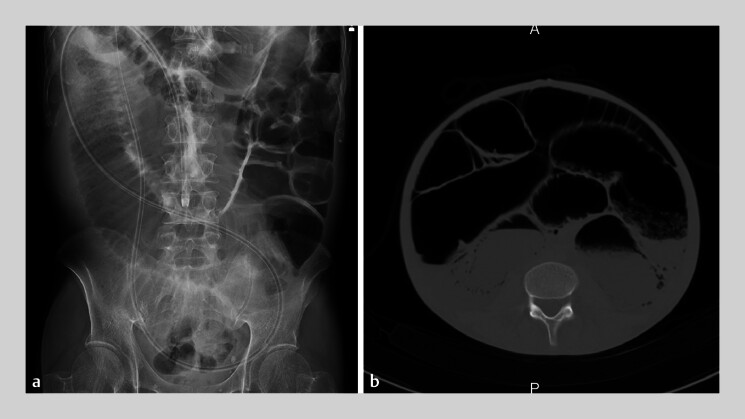
Pre-admission abdominal radiography and computed tomography images. **a** Abdominal radiograph demonstrating marked dilatation of the small intestine. **b** Computed tomography revealing significant small intestinal dilatation associated with gas-fluid levels.

Jejunal endoscopic full-thickness biopsy with the endoscopic mucosal resection using an over-the-scope clip technique.Video 1

Jejunal EFTB with EMRO was performed under sedation with midazolam, using an EI-580BT enteroscope (Fujifilm, Tokyo, Japan). A 10-mm over-the-scope clip (Type T; Ovesco Endoscopy, Tübingen, Germany) was mounted 10 mm beyond the enteroscope tip. Resection was performed using a 10-mm snare (SnareMaster Plus; Olympus, Tokyo, Japan) and an electrosurgical generator (VIO 200D; Erbe, Tübingen, Germany) set to ENDO CUT Q mode (effect: 3, cut duration: 2, and cut interval: 2). Total procedure time was 16 min, with no additional closure required. Histopathological examination confirmed full-thickness sampling, including the subserosal layer, without abnormalities in the submucosal or myenteric plexus, confirming the diagnosis of chronic idiopathic intestinal pseudo-obstruction ( Fig. 2 ).

**Fig. 2:**
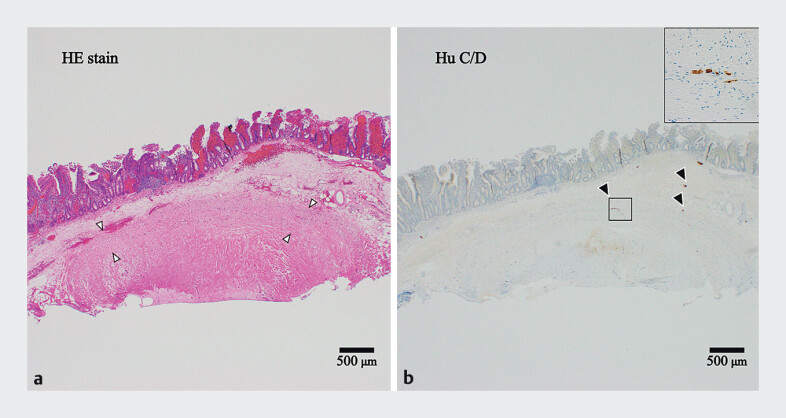
Histopathological evaluation of the resected specimen. **a** Hematoxylin and eosin staining showing full-thickness resection extending to the muscularis propria (indicated by white arrowheads). **b** HuC/D immunostaining demonstrating normally distributed and morphologically intact ganglion cells (indicated by black arrowheads), consistent with a diagnosis of chronic idiopathic intestinal pseudo-obstruction.

In patients with suspected chronic idiopathic intestinal pseudo-obstruction and unexplained obstructive symptoms, a jejunal full-thickness biopsy followed by detailed histological assessment is recommended 1 3 . Surgical full-thickness biopsy often requires adhesiolysis, with a median operative time of 50 min. Approximately 2% of patients require conversion to open surgery, and 10% are readmitted 1 . At our institution, all four patients who underwent this procedure were readmitted because of postoperative ileus. Although devices such as the full-thickness resection device (FTRD) or the “close and resect” technique have been used for EFTB 4 5 , neither has been reported for jejunal applications, and the FTRD is not approved for use in Japan. This is the first study on jejunal EFTB with EMRO, offering a promising diagnostic alternative for severe gastrointestinal motility disorders.

Endoscopy_UCTN_Code_TTT_1AP_2AD

## Notes

Endoscopy E-Videos https://eref.thieme.de/e-videos
*E-Videos* is an open access online section of the journal *Endoscopy* , reporting on interesting cases and new techniques in gastroenterological endoscopy. All papers include a high-quality video and are published with a Creative Commons CC-BY license. Endoscopy E-Videos qualify for HINARI discounts and waivers and eligibility is automatically checked during the submission process. We grant 100% waivers to articles whose corresponding authors are based in Group A countries and 50% waivers to those who are based in Group B countries as classified by Research4Life (see: https://www.research4life.org/access/eligibility/ ). This section has its own submission website at https://mc.manuscriptcentral.com/e-videos .
